# Evaluating the investment performance of Japanese households: A comparison between professional financial advice and free financial advice

**DOI:** 10.1016/j.heliyon.2025.e42213

**Published:** 2025-01-23

**Authors:** Yoshihiko Kadoya, Sumeet Lal, Jin Shinohara, Mostafa Saidur Rahim Khan

**Affiliations:** a School of Economics, Hiroshima University, 1-2-1 Kagamiyama, Higashihiroshima, 7398525, Japan; b The Investment Trusts Association of Japan, Japan

**Keywords:** Professional financial advice, Free financial advice, Financial advice from mass media, Financial advice from social media, Investment performance

## Abstract

Technological advancements have given rise to various sources of financial advice, prompting an investigation into how professional and free financial advice influence investment performance. Research lacks clarity on the impact of professional services on investment performance and overlooks the potential of free financial advice. This study, analyzing data from Rakuten Securities, a leading online-based security company in Japan, finds that investors prefer free financial advice from sources such as social media, mass media, personal networks, and individual decision-making. Regression analysis reveals that investors utilizing free financial advice from their account-holding securities company, social media, mass media, and personal networks show better investment performance than those relying on professional advice. This emphasizes a shift in investor behavior. While it is known that professional advice can lead to subpar results, our study provides new evidence that free financial advice can improve investment performance. The results suggest that professional advisors should emphasize the uniqueness and added value of their services to attract investors, particularly given the prevalence of freely available alternatives.

## 1 Introduction

The impact of financial advice on investment performance is a significant yet inconclusive issue in empirical finance. In the era of technological advancement, households often rely on a wide range of professional and free financial advice for their investment decisions [1]. The need for financial advice arises from the insufficient financial knowledge of households, which hampers their ability to navigate complex markets and makes them vulnerable to behavioral biases. With the growing participation of individual investors in financial markets, as documented by Ülkü et al. [2], Lusardi and Mitchell [3], and Barber and Odean [4], retail investors increasingly rely on financial advice to guide their decision-making in a complex and dynamic market environment. Many individuals struggle with financial decision-making and portfolio management owing to a lack of literacy [5]. Biases such as overconfidence and reliance on heuristics lead to poor economic and financial decisions, which erode wealth and reinforce the need for advice [[6], [7], [8]]. Households with lower financial literacy are more likely to seek advice when uncertain, though its quality varies [9,10]. In both the United States and Japan, low financial literacy discourages investment in risky assets, highlighting the role of advice in overcoming risk aversion and biases [11,12]. Recent research, including studies by Bhattacharya et al. [13] and Choi et al. [14], demonstrates that retail investors often benefit from financial advice; however, the quality and cost of such advice can vary significantly depending on the source. Family dynamics, such as mental health issues, further complicate financial decisions, where professional advice can provide stability [15]. Moreover, confusion about financial professionals highlights the need for clear and reliable guidance [16]. However, there is a dearth of comprehensive studies that examine how professional and free financial advice is associated with subsequent investment performance. The literature focuses predominantly on the impact of professional financial advice on investment outcomes; however, these studies have not provided conclusive evidence on the benefits of professional financial advice after accounting for commissions. Furthermore, the influence of free financial advice on investment performance has barely been explored. Against this backdrop, this study aims to investigate the association between various sources of professional and free financial advice and subsequent investment performance.

In framing the study's context, we referred to the random walk theory, which posits that stock prices are unpredictable, making it challenging for professional advisors to outperform the market [17,18] consistently. Additionally, empirical studies indicate that financial advisors are often limited by commission-driven behavior and conflicts of interest, which can lead to suboptimal product recommendations [19,20]. Moreover, advisors tend to provide reassurance rather than superior returns, often encouraging excessive trading that diminishes client performance [21]. The financial services industry is also characterized by informational asymmetries, where advisors may prioritize their own interests over clients' [22,23], and clients, as with other credence goods, are often unable to fully evaluate the quality of the advice they receive [24]. Taken together, these factors explain why the performance of professional advisors typically does not exceed market averages. Beginning from the studies of Regnault and Bachelier, many famous researchers, including Malkiel [25], Cootner [26], and Fama [27], concluded that the performance of stock market participants follows a random walk, implying that it is not possible to outperform the market consistently. In this scenario, investors might prefer to rely on free sources of information rather than costly professional advice to achieve better investment performance, at least from the perspective of cost savings.

The reasons investors seek professional financial advice and the ultimate results of such advice—whether it means maximizing returns with fixed risk or minimizing risks with fixed returns—remain unresolved. In theory, investors benefit from financial advice when the returns from their investments significantly exceed the cost of obtaining information [28,29]. However, the literature presents a divided perspective on the results of professional financial advice. Studies have found that professional financial advice struggles to outperform the market, particularly when considering net returns after accounting for commissions [[17], [18], [19], [20], [21], [22]]. By contrast, alternative studies suggest that professional financial advice occasionally contributes to superior portfolio performance [[30], [31], [32], [33], [34], [35], [36]]. For example, Warren Buffett's active strategy outperformed the stock market and demonstrated the potential for skill-based active management [37]. However, such cases are rare, and most professional advisors do not achieve these results. Studies asserting that professional financial advice does not guarantee superior investment performance often highlight agency costs or profit-seeking motives of financial advisors as the underlying reasons for subpar financial outcomes [[19], [20], [21], [22], [23]]. The agency problem arises from the dual roles that advisors must fulfill: selling financial products and advising customers. The conflicting nature of these roles, compounded by commissions and fees, can lead to portfolio losses. For instance, Hackethal et al. [21] suggested that advisors may be aware of customers' best interests; however, their incentive to mislead uninformed customers for personal gain can result in information asymmetry. This information asymmetry is particularly problematic when customers lack financial knowledge and trust advisors without having control, ultimately leading to portfolio losses [13,22,33,38,39]. However, reputation and historical portfolio performance can serve as indicators of advisor quality, playing a crucial role in reducing information asymmetry [33,38]. While these factors can reduce information asymmetry, the fundamental issue remains that clients often lack sufficient financial knowledge and control, which can lead to significant portfolio losses even when relying on advisors with strong reputations and proven track records [40,41].

The uncertain performance of professional financial advice, coupled with technological advancements, has led many investors to seek financial advice from free sources of financial advice such as social media, mass media, or online financial experts [1]. Recent studies by Ülkü et al. [2], Hryckiewicz and Kozłowski [42], and Foerster et al. [43] suggest that individual investors are increasingly relying on technology-based free advisory services in their financial decision-making process, often bypassing traditional professional advice. Lal et al. [1] conducted a study involving nearly 65,000 active investors in Japan to explore the information sources they considered valuable for their investments. Their findings revealed that active investors showed a greater inclination toward recently emerged technology-driven sources, such as social media and mass media, as opposed to traditional sources such as financial advisors or investment groups. The study further highlighted that social and mass media were particularly popular among young investors, while older investors tended to prefer financial advisor guidance. The significant role of technology in the financial decision-making process cannot be underestimated. Social media platforms such as Facebook, Instagram, and YouTube have played a crucial role in shaping opinions within the financial market [[44], [45], [46]]. However, the performance of investments following the widespread dissemination of information through social media or opinions provided by financial experts online without fees has rarely been investigated on a large scale.

Considering the inconclusive evidence on the necessity of professional financial advice and the lack of studies on the impact of free financial advice on subsequent investment performance, a comprehensive study is imperative. This study aims to evaluate subjective investment performance resulting from both professional financial advice and free financial advice in Japan. Our study contributes to the existing literature in at least two significant ways. First, we examine investors’ preferences for professional versus free financial advice and assess the impact of such advice on subsequent investment performance. Second, utilizing a large-scale, recent dataset of active investors in Japan, we offer evidence on whether the demand for professional financial advice is decreasing. This evidence can be instrumental in validating claims related to agency problems or profit-seeking motives of securities companies associated with financial advice.

The remainder of the paper is organized as follows: Section 2 outlines the data and methodology, Section 3 presents the empirical findings of the study, Section 4 discusses the findings and compares them with previous studies, and Section 5 concludes.

## 2 Literature review

The financial markets have experienced transformative changes with the increasing prominence of individual (retail) investors, who now significantly impact market dynamics. Historically, institutional investors, supported by extensive research and dedicated investment teams, dominated financial markets. However, advancements in technology and the proliferation of online trading platforms have democratized access to financial markets, leading to a significant increase in individual investor participation [47,48]. Moreover, Ülkü et al. [2] conducted a detailed analysis of the increase in individual investor activity during the COVID-19 pandemic, identifying key factors driving this trend. Their study highlights two primary influences: (i) unprecedented monetary and fiscal policy measures implemented during the pandemic, and (ii) the lockdowns and the shift to remote work, which provided individuals with more free time to engage in financial trading. The extensive government stimulus packages and low interest rates created a favorable environment for retail trading. Concurrently, the lockdowns and remote work arrangements enabled individuals to actively monitor and trade financial markets, leading to increased engagement. This heightened activity revealed a level of market involvement that challenges previous assumptions about retail investor sophistication and highlights their growing influence on market dynamics. Further supporting this trend, Crook et al. [49] found that retail investors are particularly attracted to “attention-grabbing stocks” and are more likely to react to high-profile events during periods of uncertainty. Additionally, Chiah and Zhong [50] observed that news events during the pandemic significantly influenced retail investing behaviors, contributing to increased trading volumes. Baig et al. [51] investigated the relationship between retail trading and market volatility during the pandemic, revealing that increased volatility often coincided with increased retail trading activity, particularly before the pandemic. Thus, the increased involvement of individual investors has resulted in a shift in the demand and nature of financial advice [52]. Retail investors now have access to a wider range of advisory services, including free advice from social media and mass media platforms and AI-driven tools, in addition to traditional professional advice [53]. This shift highlights the need for a comprehensive understanding of how different sources of advice influence individual investment performance, particularly as these investors become more influential in market movements.

The sources of information for investment decisions are diverse and have evolved significantly over time. Historically, professional financial advice from experts and institutions has played a crucial role in guiding investors. However, the performance of such advice has not consistently surpassed the market average. Studies have demonstrated that professional financial advisors often struggle to outperform market indices in the long term [54]. This is consistent with the efficient market hypothesis (EMH) and random walk theory, which suggest that consistently outperforming the market through expert advice alone is unlikely. The EMH, proposed by Fama [27], posits that financial markets are efficient and that asset prices reflect all available information. Consequently, it is challenging for any investor, including professionals, to achieve returns that consistently exceed market averages [55]. Similarly, the random walk theory, popularized by Malkiel [25], argues that stock prices move unpredictably and that past price movements cannot be used to predict future prices. Both theories imply that professional financial advice cannot reliably outperform the market in the long run.

The emergence of digital platforms has led to the proliferation of free financial advice through sources such as social media, mass media, and other online forums [56,57]. These platforms are popular among younger investors who are more technologically savvy. Several studies have demonstrated how media, including social media and traditional print media, improve financial decision-making by facilitating access to information, filtering information, enhancing information coverage diversity, and creating new information [[58], [59], [60], [61]]. These studies suggest that the media, acting as an information intermediary, contributes to market efficiency, higher investor returns, and lower costs for firms. Moreover, recent technological advancements, particularly in artificial intelligence (AI), have democratized access to free financial information. AI-driven tools and platforms can enhance investors’ decision-making by offering diverse perspectives and real-time updates [62,63]. However, despite their widespread use, the accuracy and reliability of free financial advice are often questioned. Studies by Barberis et al. [64] and Vasquez and Cross [65] indicate that while free advice can offer valuable insights, it may also be prone to biases, misinformation, or misinterpretation.

Although the accuracy of free advice from sources such as mass media, social media, or AI-generative tools is sometimes questioned in studies, it can effectively complement professional advice when used by prudent investors [66]. Integrating free and professional advice might offer a balanced approach, leveraging the strengths of both sources while mitigating their respective limitations. Olajide et al. [67] found that using various social media platforms influences financial satisfaction. Because financial professionals highly value clients' involvement and satisfaction, they can use social media platforms’ widespread popularity to create content that resonates with different generations, thereby expanding their audience reach. Additionally, Reiter [68] investigated the characteristics of consumers using social media as an information source for investment advice and suggested that social media can facilitate engagement and information exchange, thereby complementing professional advice. Similarly, several studies have explored how AI can streamline processes, provide real-time data analysis, and offer predictive analytics, which can complement the personalized advice provided by human advisors across the wealth management sector [69,70]. Furthermore, recent research further highlights the evolving expectations of advised clients in the financial sectors. According to Brin [52], research by Cerulli Associates and the Securities Industry and Financial Markets Association shows that clients value the reassurance of having dedicated financial advisors and are keen on solutions from trusted providers. While digital tools and AI offer new opportunities, they should complement rather than replace human advisors.

Nonetheless, despite the growing body of literature on various sources of financial advice and investor behavior, a notable research gap persists in understanding how different sources of financial information directly impact subjective portfolio performance. Most existing studies have focused on the efficacy of professional financial advisors or have examined the general impact of free financial information without linking it explicitly to subjective performance outcomes. This gap in the literature highlights the need for further research to understand how different sources of financial advice influence individual investors’ perceptions of their portfolio performance. Addressing this gap could provide valuable insights into optimizing the use of diverse information sources to enhance investment outcomes.

## 3 Materials and methods

### 3.1 Data

This study utilized large-scale data from the 2023 wave of the “Survey on life and money,” an online survey conducted by Rakuten Securities, one of Japan's largest online security companies, in collaboration with Hiroshima University. Their database covers all socioeconomic segments of the Japanese population and is one of the largest in the country. The survey, conducted in November and December 2023, targeted active account holders of the securities company who were 18 years or older and lived in Japan. The survey included questions about the demographic, socioeconomic, psychological, and preferences of Japanese adults. Only the 2023 wave had questions on detailed financial information sources for investors and their investment performances. After removing missing data, the sample size was 184,698 observations, representing 65.37 % of the valid responses from 282,546 observations. Therefore, the final data of this study appeared adequate and representative enough to provide unbiased results.

This study was based on a socio-economic survey, which did not involve any aspect of a direct human experiment. Moreover, all data used were anonymized. The authors obtained informed consent from the participants to participate in the survey and to publish the survey data in the study.

### 3.2 Variables

Our primary dependent variable assesses subjective investment performance, measured on a 5-point scale derived from the question: “Please indicate the profit and loss status of the investment in financial securities.” The respondents selected from five options: (1) significant profit, (2) small profit, (3) almost balanced profit and loss, (4) small loss, and (5) significant loss. To have robust subjective investment performance, we created a binary variable: 1 indicating a significant or small loss from investment and 0 representing other outcomes.

Our primary independent variables include various financial advisory sources selected by investors. We assessed investors’ preference for specific financial advice sources using the survey question: “Who do you primarily rely on for advice regarding asset management (investment)?” The respondents had eight options but could only select one current source. The choices included (i) advice from the outsourced independent financial advisors of the account-holding securities company, (ii) free advice from the account-holding securities company, (iii) advice from other securities companies, (iv) advice from experts apart from securities companies, (v) advice from mass media, (vi) advice from social media, (vii) advice from personal networks such as colleagues, family members, and friends, and (viii) self-decision. Among the sources, advice from outsourced independent financial advisors of the account-holding securities company, other securities companies, and experts outside the securities companies is considered professional advice, which is chargeable, whereas free advice from the account-holding securities company, financial advice through mass media, social media, and personal networks is categorized as free financial advice.

Finally, we control demographic and socioeconomic factors to isolate the independent impact of financial advice on investment performance. Our study used several control variables: gender, age, marital status, having children, employment status, education, urban residency, household income, household assets, risk aversion, and myopic view of the future. Table 1 provides detailed definitions and measurements of the dependent, independent, and control variables.

**Table 1:** Variable definitions.

Dependent variable	
Investment_status	Ordinal variable: Subjective investment performance of the respondents, measured on a five-point scale where 1 indicates a significant profit and 5 indicates a significant loss.
Investment_status_loss	Binary variable: 1 indicates that the respondents experienced a significant or small loss in their investments, and 0 otherwise
Independent variables	
Own_decision (base)	Binary variable: 1 indicates the investor's preference for self-decision, and 0 otherwise
Financial advice_ outsourced IFA of the securities company	Binary variable: 1 indicates investors' preference for financial advice from the outsourced “independent financial advisor (IFA)” of the securities company where they maintained investment accounts, and 0 otherwise
Financial advice_free from the securities company	Binary variable: 1 indicates the investor's preference for free financial advice from the securities company where they maintained investment accounts, and 0 otherwise
Financial advice_other securities companies	Binary variable: 1 indicates investors' preference for financial advice from other securities companies, and 0 otherwise
Financial advice_financial experts apart from securities companies	Binary variable: 1 indicates investors' preference for financial advice from financial experts apart from the security companies, and 0 otherwise
Financial advice_mass media	Binary variable: 1 indicates investors' preference for financial information from mass media (newspaper, magazines and television), and 0 otherwise
Financial advice_social media	Binary variable: 1 indicates investors' preference for financial information from blogs, YouTube, Instagram, etc., and 0 otherwise
Financial advice_personal networks	Binary variable: 1 indicates investors' preference for financial advice from close associates such as family, friends, and colleagues, and 0 otherwise
Male	Binary variable: Equal to 1 if the respondents are males
Age	Continuous variable: age of the respondents
Age squared	Continuous variable: Square of each age
University degree	Binary variable: Equal to 1 if the respondents hold at least a university degree, 0 otherwise
Urban residency	Binary variable: Equal to 1 if the respondents reside in an urban area, 0 otherwise
unemployed	Binary variable: Equal to 1 if the respondents are unemployed
Financial literacy	Continuous variable: average score for number of correct answers from the three financial literacy questions
Married	Binary variable: Equal to 1 if the respondent is married, 0 = otherwise
Have children	Binary variable: Equal to 1 if the respondent at least has a child, 0 = otherwise
Household income	Continuous variable: Respondents' income measured in Japanese Yen
Log of household income	Continuous variable: Natural log of the respondents' income
Household assets	Continuous variable: Household financial assets measured in Japanese Yen
Log of household assets	Continuous variable: Natural log of the respondents' household financial assets
Risk aversion	Continuous variable: risk of rain preference (percentage score from the question, “Usually when you go out, how high must the probability of rainfall be before you take an umbrella?”)
Myopic view of the future	Binary variable: Equal to 1 if respondents agree or completely agree with, “Since the future is uncertain, it is a waste to think about it,” 0 = otherwise

### 3.3 Robustness check

To enhance the robustness of our findings, we performed additional analyses using the propensity score matching (PSM) method, specifically applying the nearest neighbor matching technique/algorithm for the ordinal measure of subjective investment performance (outcome variable). Propensity scores were estimated separately for each treatment variable (independent variable) using probit regression, incorporating covariates such as age, gender, marital status, having children, employment status, education, urban residency, household income, household assets, risk aversion, and myopic view of the future. A block identifier was employed to organize and streamline the matching process by ensuring comparisons within groups of subjects with similar propensity scores, leading to more accurate and reliable treatment effect estimates. Within the PSM, the nearest neighbor matching technique was performed using bootstrap replications and the common support option to confirm that the matched samples were within the overlapping region of propensity scores for both treated and control units. To ensure well-balanced covariates, blocks with significant imbalances were excluded, thereby enhancing the overall balance between the treated (those who used the specified source of information) and control groups (those who did not use the specified source of information). Finally, a regression model was re-estimated using the matched sample for each treated group.

### 3.4 Descriptive statistics

Table 2 presents descriptive statistics for the study. The results indicate that 16.52 % of the respondents achieved a substantial profit, 64.61 % earned a modest profit, 9.54 % broke even, 5.23 % experienced a slight loss, and 4.10 % experienced a significant loss. Another metric for investment status reveals that 9.33 % of the respondents reported experiencing varying degrees of loss in their investments. Regarding sources of financial advice, 1.71 % of the respondents relied on the outsourced IFA of the account-holding securities company, 8.55 % on free financial advice from the account-holding securities company, 2.57 % on other securities companies, 3.53 % on financial experts apart from securities companies, 13.29 % in mass media, 33.39 % on social media, 9.15 % on personal networks, and 27.63 on their decision-making. Overall, the respondents predominantly leaned toward free financial advice or self-decision, with limited reliance on professional financial advice.

**Table 2:** Descriptive statistics.

	Mean/Frequency	SD	Min	Max
Dependent variables				
Investment_status (ordinal)				
Quite profitable	0.1652	–	0	1
Slightly profitable	0.6461	–	0	1
Breakeven	0.0954	–	0	1
Slightly loss	0.0523	–	0	1
Significantly loss	0.0410	–	0	1
Investment_status (Binary)				
Loss	0.0933	–	0	1
Otherwise	0.9067	–	0	1
Independent variables				
Financial advice_ outsourced IFA of securities company	0.0171	–	0	1
Financial advice_free from securities company	0.0855	–	0	1
Financial advice_other securities companies	0.0257	–	0	1
Financial advice_financial experts apart from securities companies	0.0353	–	0	1
Financial advice_mass media	0.1329	–	0	1
Financial advice_social media	0.3339	–	0	1
Financial advice_personal networks	0.0915	–	0	1
Own_decision	0.2763	–	0	1
Control variables				
Age	45.2726	12.2672	18	94
Age Squared	2200.0870	1167.3180	324	8836
Age Group				
18–29	0.101411	0.301872	0	1
30–39	0.256728	0.436829	0	1
40–49	0.279816	0.44891	0	1
50–59	0.220166	0.414359	0	1
60+	0.141881	0.348929	0	1
Male	0.6496	0.4771	0	1
University Degree	0.6445	0.4787	0	1
Urban Residency	0.1590	0.3657	0	1
Unemployment	0.0642	0.2451	0	1
Financial Literacy	0.7919	0.2975	0	1
Married	0.6714	0.4697	0	1
Have Children	0.5561	0.4968	0	1
Household Income	5.2420	2.1627	1	12
Log of Household Income	15.6667	0.6076	13.8155	16.8112
Household Asset	4.5721	2.6955	1	10
Log of Household Asset	16.1818	1.0856	14.7318	18.4206
Risk Aversion	0.5358	0.2309	0	1
Myopic view of the future	0.1519	0.3589	0	1
N– = 184,698				

Additionally, Table 3 illustrates the distribution of each information source across age groups. As suggested by Lal et al. [1], information sources exhibit generation-dependent trends, showcasing the sources preferred by individuals in each age bracket. Our study, using ANOVA tests, confirms statistically significant differences in preference for financial advisory sources between generations. The results reveal that younger and middle-aged investors leaned more toward social media than their older counterparts, while older investors showed a greater reliance on mass media compared with the younger and middle-aged groups. Additionally, across all age groups, a common trend was observed in which investors tended to rely less on professional financial advice, particularly those involving fees.

**Table 3:** The distribution of financial advisory sources by age group.

Information Sources			Age Group			
	18–29	30–39	40–49	50–59	60+	Total
Financial advice_outsourced IFA of securities company	510	894	890	572	295	3161
%	3.11	2.07	1.84	1.49	1.19	1.85
Financial advice_free from securities company	1076	2932	4356	4209	3213	15,786
%	6.57	6.79	9.00	10.98	12.98	9.23
Financial advice_other securities companies	467	1004	1194	1153	924	4742
%	2.85	2.33	2.47	3.01	3.73	2.77
Financial advice_financial experts apart from securities companies	608	1733	1912	1409	853	6515
%	3.71	4.01	3.95	3.68	3.45	3.81
Financial advice_mass media	1638	4308	6120	6662	5801	24,529
%	10	9.98	12.64	17.38	23.43	14.34
Financial advice_social media	7121	19,418	18,168	11,928	5008	61,643
%	43.46	44.97	37.53	31.12	20.23	36.04
Financial advice_personal networks	639	1186	1102	548	178	3653
%	3.90	2.75	2.28	1.43	0.72	2.14
Own Decision	4325	11,702	14,664	11,843	8487	51,021
%	26.40	27.10	30.29	30.90	34.28	29.83
Total	16,384	43,177	48,406	38,324	24,759	171,050
%	100	100	100	100	100	100

F = 86.22∗∗∗.

Regarding socioeconomic factors, male investors comprised 64.96 % of the total. Regarding age distribution, the percentages in each age group were as follows: 10 % in their 20s, 26 % in their 30s, 28 % in their 40s, 22 % in their 50s, and 14 % in their 60s and above. Furthermore, 64.45 % of the respondents had a university degree, 15.90 % resided in urban areas, 6 % were unemployed, 67 % were married, and 55.61 % had children. Additionally, the respondents’ average income and household assets were JPY 5,242,000 and JPY 45,721,000, respectively. Finally, 53.58 % of the respondents exhibited risk aversion, and 15.19 % had a myopic view of the future.

### 3.5 Methods

To investigate the relationship between financial advisory sources and subjective investment performance, we employed the following estimation equations:

(1) $$Y_{1i}=f\left(F{AS}_{i},X_{i},\varepsilon_{i}\right)$$

(2) $$Y_{2i}=f\left({FAS}_{i},X_{i},\varepsilon_{i}\right)$$

where $Y_{1i}$ and $Y_{2i}$ are the measures of the dependent variables, “Investment-status” and “Investment-status_Loss” respectively. $F{AS}_{i}$ indicates various financial advisory sources, $X_{i}$ indicates vectors of an individual's socioeconomic and demographic status and $\varepsilon_{i}$ is the error term.

We evaluated the investment status using two methods. Initially, it was evaluated on a five-point scale, where 5 indicated a substantial loss and 1 indicated a significant profit. Subsequently, we transformed this scale into a dichotomous response, with 1 representing a loss and 0 otherwise. Therefore, for these two measures of investment performance, we used ordered probit models and probit models to estimate Equations (1), (2), respectively. While both probit and logit models could be used for binary outcomes, we opted for the probit model because it assumes a normal distribution of errors, which aligns with our assumptions about the distribution of the latent variable influencing investment outcomes. Moreover, our choice for the probit model is supported by its application in similar financial studies. For instance, Korniotis and Kumar [71] utilized the probit model to explore the influence of age on investment decisions and portfolio performance, while Christelis et al. [72] applied it to assess the impact of cognitive abilities on portfolio choices, demonstrating its effectiveness in financial decision contexts.

We used our own decision as the base outcome of the model. The primary independent variables include various sources of financial advice, such as financial advice from the outsourced IFA of the account-holding securities company, free financial advice from the account-holding company, financial advice from other financial institutions, financial advice from the financial experts apart from securities companies, financial advice from the mass media and social media, and personal networks. The demographic and socioeconomic control variables remained consistent in all specifications.

We examined the variance inflation factors (VIF) to explore the potential presence of multicollinearity. Our findings revealed no indication of multicollinearity across all models, as the VIF were below 10 (results not reported here owing to space constraints but available upon request). The following are the complete specifications for Equations (1), (2):

[3] $${Investment-status}_{i}=\beta_{0}+\beta_{1}{\text{Financial}\;\text{advice}\_\;\text{outsourced}\;\text{IFA}\;\text{of}\;\text{securites}\;\text{company}}_{i}+\beta_{2}{\text{Financial}\;\text{advice}\_\;\text{free}\;\text{from}\;\text{securites}\;\text{company}}_{i}+\beta_{3}{\text{Financial}\;\text{advice}\_\text{other}\;\text{securities}\;\text{companies}}_{i}+\beta_{4}{\text{Financial}\;{\text{advice}}_{-}\text{financial}\;\text{experts}\;\text{apart}\;\text{from}\;\text{securities}\;\text{companies}}_{i}+\beta_{5}{\text{Financial}\;\text{advice}\_\text{mass}\;\text{media}}_{i}+\beta_{6}{\text{Financial}\;\text{advice}\_\text{social}\;\text{media}}_{i}+\beta_{7}{\text{Financial}\;\text{advice}\_\text{personal}\;\text{networks}}_{i}+\beta_{8}{age}_{i}+\beta_{9}{age}_{i}^{2}+\beta_{10}{age\_30s}_{i}+\beta_{11}{age\_40s}_{i}+\beta_{12}{age\_50s}_{i}+\beta_{13}{age\_60s}_{i}+\beta_{14}{male}_{i}+\beta_{15}{university\;degree}_{i}+\beta_{16}{urban\;residency}_{i}+\beta_{17}{unemployed}_{i}+\beta_{18}{married}_{i}+\beta_{19}{have\;children}_{i}+\beta_{20}{financial\;literacy}_{i}+\beta_{21}{\ln\;\_income}_{i}+\beta_{22}{\ln\;\_household\_assets}_{i}+\beta_{23}{risk\;aversion}_{i}+\beta_{24}{myopic\;view\;of\;the\;future}_{i}+\varepsilon_{i}$$

[4] $$Investment\_Status\_{Loss}_{i}\left(1=significant\;or\;small\;loss\;and\;0=otherwise\right)=\beta_{0}+\beta_{1}{\text{Financial}\;\text{advice}\_\;\text{outsourced}\;\text{IFA}\;\text{of}\;\text{securites}\;\text{company}}_{i}+\beta_{2}{\text{Financial}\;\text{advice}\_\;\text{free}\;\text{from}\;\text{securites}\;\text{company}}_{i}+\beta_{3}{\text{Financial}\;\text{advice}\_\text{other}\;\text{securities}\;\text{companies}}_{i}+\beta_{4}{\text{Financial}\;\text{advice}\_\text{financial}\;\text{experts}\;\text{apart}\;\text{from}\;\text{securities}\;\text{companies}}_{i}+\beta_{5}{\text{Financial}\;\text{advice}\_\text{mass}\;\text{media}}_{i}+\beta_{6}{\text{Financial}\;\text{advice}\_\text{social}\;\text{media}}_{i}+\beta_{7}{\text{Financial}\;\text{advice}\_\text{personal}\;\text{networks}}_{i}+\beta_{8}{age}_{i}+\beta_{9}{age}_{i}^{2}+\beta_{10}{age\_30s}_{i}+\beta_{11}{age\_40s}_{i}+\beta_{12}{age\_50s}_{i}+\beta_{13}{age\_60s}_{i}+\beta_{14}{male}_{i}+\beta_{15}{university\;degree}_{i}+\beta_{16}{urban\;residency}_{i}+\beta_{17}{unemployed}_{i}+\beta_{18}{married}_{i}+\beta_{19}{have\;children}_{i}+\beta_{20}{financial\;literacy}_{i}+\beta_{21}{\ln\;\_income}_{i}+\beta_{22}{\ln\;\_household\_assets}_{i}+\beta_{23}{risk\;aversion}_{i}+\beta_{24}{myopic\;view\;of\;the\;future}_{i}+\varepsilon_{i}$$

## 4 Results

We conducted a cross-sectional regression analysis utilizing ordered probit and probit models to assess the relationship between advisory sources and subjective investment performance. As outlined in the methodology section, we introduced two types of dependent variables to capture different aspects of investment performance: the five-point scale (used for ordered probit models) was employed to measure the degree of loss or profit, and the dichotomous variable (used for probit models) was utilized to distinguish between loss and non-loss outcomes. This dual approach enables a more comprehensive analysis of investment outcomes. Table 4 presents the regression results derived from the ordered probit model, whereas Table 5 provides the results from the probit model. Table 4, Table 5 present the four specifications. Model 1 comprises only financial advisory sources, Model 2 incorporates demographic features in addition to Model 1, Model 3 integrates economic status along with Model 2, and Model 4 represents the complete model encompassing all explanatory variables.

**Table 4:** Ordered probit regression results.

Variables	Model 1	Model 2	Model 3	Model 4
Financial advice_ from outsourced IFA of securities company	0.0580∗∗∗	0.0885∗∗∗	−0.0138	−0.0139
	(0.0205)	(0.0206)	(0.0211)	(0.0211)
Financial advice_ free from securities company	−0.0244∗∗	−0.0263∗∗∗	−0.0493∗∗∗	−0.0492∗∗∗
	(0.0099)	(0.0100)	(0.0100)	(0.0100)
Financial advice_ other securities companies	−0.0114	−0.0002	0.0378∗∗	0.0380∗∗
	(0.0176)	(0.0177)	(0.0178)	(0.0178)
Financial advice_ financial experts apart from securities companies	−0.0382∗∗	−0.0126	−0.0209	−0.0208
	(0.0152)	(0.0152)	(0.0153)	(0.0153)
Financial advice_ mass media	−0.0397∗∗∗	−0.0427∗∗∗	−0.0201∗∗	−0.0199∗∗
	(0.0089)	(0.0089)	(0.0089)	(0.0089)
Financial advice_ social media	−0.2686∗∗∗	−0.2372∗∗∗	−0.2350∗∗∗	−0.2348∗∗∗
	(0.0069)	(0.0070)	(0.0071)	(0.0071)
Financial advice_ personal networks	−0.0572∗∗∗	−0.0020	−0.0455∗∗∗	−0.0454∗∗∗
	(0.0093)	(0.0096)	(0.0098)	(0.0098)
Age		−0.0070∗∗	0.0142∗∗∗	0.0142∗∗∗
		(0.0031)	(0.0031)	(0.0031)
Age squared		0.0001∗∗∗	−0.0000	−0.0000
		(0.0000)	(0.0000)	(0.0000)
30–39 Years		0.0528∗∗∗	0.0799∗∗∗	0.0798∗∗∗
		(0.0148)	(0.0150)	(0.0150)
40–49 Years		0.0894∗∗∗	0.1171∗∗∗	0.1172∗∗∗
		(0.0234)	(0.0238)	(0.0238)
50–59 Years		0.0717∗∗	0.1146∗∗∗	0.1147∗∗∗
		(0.0294)	(0.0299)	(0.0299)
60+ Years		0.0269	0.0811∗∗	0.0812∗∗
		(0.0354)	(0.0358)	(0.0358)
Male		−0.0657∗∗∗	−0.0946∗∗∗	−0.0945∗∗∗
		(0.0056)	(0.0058)	(0.0058)
University degree		−0.1566∗∗∗	−0.0062	−0.0064
		(0.0057)	(0.0060)	(0.0060)
Urban residency		−0.0388∗∗∗	0.0064	0.0062
		(0.0073)	(0.0073)	(0.0073)
unemployed		0.0760∗∗∗	0.0613∗∗∗	0.0614∗∗∗
		(0.0135)	(0.0138)	(0.0138)
Married		−0.0692∗∗∗	0.0379∗∗∗	0.0380∗∗∗
		(0.0069)	(0.0074)	(0.0074)
Have children		0.0044	−0.0169∗∗	−0.0166∗∗
		(0.0067)	(0.0067)	(0.0067)
Financial literacy			−0.2371∗∗∗	−0.2368∗∗∗
			(0.0092)	(0.0092)
Log of household income			−0.1316∗∗∗	−0.1315∗∗∗
			(0.0059)	(0.0059)
Log of household assets			−0.2195∗∗∗	−0.2196∗∗∗
			(0.0031)	(0.0031)
Risk aversion				0.0121
				(0.0120)
Myopic view of the future				0.0113
				(0.0075)
/cut1	−1.0833∗∗∗	−1.2702∗∗∗	−6.3419∗∗∗	−6.3316∗∗∗
	(0.0058)	(0.0634)	(0.1036)	(0.1037)
/cut2	0.7869∗∗∗	0.6136∗∗∗	−4.3880∗∗∗	−4.3777∗∗∗
	(0.0055)	(0.0633)	(0.1030)	(0.1031)
/cut3	1.2290∗∗∗	1.0620∗∗∗	−3.9266∗∗∗	−3.9164∗∗∗
	(0.0059)	(0.0633)	(0.1028)	(0.1029)
/cut4	1.6498∗∗∗	1.4887∗∗∗	−3.4923∗∗∗	−3.4821∗∗∗
	(0.0068)	(0.0633)	(0.1028)	(0.1029)
Observations	184,698	184,698	184,698	184,698
Log-likelihood	−200100	−198964	−193928	−193926
Chi2 statistics	2030	4319	13248	13251
p-value	0	0	0	0

Note: Robust standard errors in parentheses. ∗∗∗p < 0.01, ∗∗p < 0.05, ∗p < 0.10.

**Table 5:** Probit regression results.

Variables	Model 1	Model 2	Model 3	Model 4
Financial advice_ from outsourced IFA of securities company	−0.0730∗∗	−0.0013	−0.0668∗∗	−0.0650∗∗
	(0.0307)	(0.0311)	(0.0317)	(0.0317)
Financial advice_ free from securities company	−0.1438∗∗∗	−0.1367∗∗∗	−0.1544∗∗∗	−0.1528∗∗∗
	(0.0156)	(0.0159)	(0.0160)	(0.0160)
Financial advice_ other securities companies	−0.0462∗	−0.0212	0.0085	0.0106
	(0.0251)	(0.0255)	(0.0258)	(0.0258)
Financial advice_ financial experts apart from securities companies	−0.0621∗∗∗	−0.0030	−0.0031	−0.0008
	(0.0219)	(0.0221)	(0.0224)	(0.0224)
Financial advice_ mass media	−0.0963∗∗∗	−0.1098∗∗∗	−0.0942∗∗∗	−0.0922∗∗∗
	(0.0130)	(0.0133)	(0.0134)	(0.0134)
Financial advice_ social media	−0.3223∗∗∗	−0.2476∗∗∗	−0.2439∗∗∗	−0.2429∗∗∗
	(0.0105)	(0.0108)	(0.0110)	(0.0110)
Financial advice_ personal networks	−0.2851∗∗∗	−0.1283∗∗∗	−0.1534∗∗∗	−0.1528∗∗∗
	(0.0161)	(0.0169)	(0.0172)	(0.0172)
Age		0.0052	0.0186∗∗∗	0.0188∗∗∗
		(0.0050)	(0.0052)	(0.0052)
Age squared		0.0001	−0.0000	−0.0000
		(0.0000)	(0.0000)	(0.0000)
30–39 Years		0.0780∗∗∗	0.0916∗∗∗	0.0911∗∗∗
		(0.0275)	(0.0277)	(0.0277)
40–49 Years		0.1030∗∗	0.1147∗∗∗	0.1145∗∗∗
		(0.0422)	(0.0428)	(0.0428)
50–59 Years		0.0741	0.0942∗	0.0945∗
		(0.0521)	(0.0528)	(0.0528)
60+ Years		0.0268	0.0435	0.0447
		(0.0594)	(0.0604)	(0.0604)
Male		−0.2654∗∗∗	−0.2801∗∗∗	−0.2807∗∗∗
		(0.0098)	(0.0101)	(0.0101)
University degree		−0.1727∗∗∗	−0.0683∗∗∗	−0.0676∗∗∗
		(0.0088)	(0.0092)	(0.0093)
Urban residency		−0.0131	0.0199∗	0.0201∗
		(0.0115)	(0.0117)	(0.0117)
unemployed		0.1166∗∗∗	0.0783∗∗∗	0.0784∗∗∗
		(0.0177)	(0.0186)	(0.0186)
Married		−0.1015∗∗∗	−0.0149	−0.0147
		(0.0108)	(0.0116)	(0.0116)
Have children		−0.0125	−0.0200∗	−0.0195∗
		(0.0105)	(0.0107)	(0.0107)
Financial literacy			−0.1123∗∗∗	−0.1108∗∗∗
			(0.0146)	(0.0147)
Log of household income			−0.1286∗∗∗	−0.1282∗∗∗
			(0.0088)	(0.0088)
Log of household assets			−0.1439∗∗∗	−0.1433∗∗∗
			(0.0047)	(0.0047)
Risk aversion				−0.0030
				(0.0185)
Myopic view of the future				0.0559∗∗∗
				(0.0115)
Constant	−1.1700∗∗∗	−1.1624∗∗∗	2.7424∗∗∗	2.7116∗∗∗
	(0.0072)	(0.1055)	(0.1630)	(0.1632)
Observations	184,698	184,698	184,698	184,698
Log-likelihood	−56690	−55243	−54247	−54235
Chi2 statistics	1090	3820	5264	5284
p-value	0	0	0	0

Note: Robust standard errors in parentheses. ∗∗∗p < 0.01, ∗∗p < 0.05, ∗p < 0.10.

Table 4 presents the regression results derived from the ordered probit model, utilizing subjective investment status as an ordinal variable. The results indicate a negative association between subjective investment status and free financial advice from the account-holding securities company, mass media, social media, and personal networks. This suggests that investors who predominantly rely on advice from these sources have made significant profits in their investments. By contrast, investors who relied on financial experts from other securities companies experienced significant losses. There appears to be no significant association between investment performance and professional advice from the IFA of the account-holding securities company and financial experts apart from securities companies. Across all control variables, investors tended to experience losses as they aged. The insignificance of the age-squared variable confirms that the relationship between investment performance and age remains consistent across various stages of life. Furthermore, married and unemployed investors also experienced losses. However, investors who are male, as well as those with children, financial literacy, higher income, and larger assets, made significant profits.

Table 5 presents the regression results of the probit model, using the subjective investment status as a binary variable to check the robustness of the results. The results indicate a negative association between subjective investment status and professional advice from the IFA of the account-holding securities company, free financial advice from the account-holding securities company, mass media, social media, and personal networks. This suggests that investors who predominantly rely on advice from these sources have made significant profits in their investments. However, there appears to be no significant association between investment performance and financial advice from securities companies and financial experts apart from securities companies. Across all control variables, investors tended to experience losses as they aged. The insignificance of the age-squared variable confirms that the relationship between investment performance and age remains consistent across various stages of life. Furthermore, investors who were unemployed, had an urban residency, and had a myopic view of the future also experienced losses. However, investors who were male, with a university degree, children, financial literacy, higher income, and greater asset holdings made significant profits.

Table 6 presents the results of the re-estimated regression models for each primary independent variable, utilizing the matched sample based on the ordinal measure of subjective investment performance. This table includes seven comprehensive model specifications, each representing a distinct information source and incorporating a complete set of relevant demographic and socioeconomic variables. The robustness test validates the initial findings and confirms the reliability of the treatment effect estimates for each explanatory variable. The consistency across analyses reveals that the negative association between subjective investment status and reliance on free financial advice from the account-holding securities company, mass media, social media, and personal networks remains significant. Similarly, the adverse effect of financial advice from experts by other securities companies on investment performance was upheld. Furthermore, the lack of significant association between investment performance and professional advice from the IFA of the account-holding securities company or financial experts, apart from securities companies, was consistently observed. Among the control variables and consistent across all models, older investors experienced losses, while married and unemployed investors faced financial setbacks. By contrast, male investors with higher financial literacy, increased income, and larger assets continued to achieve significant profits. These results align with the main findings, reinforcing the reliability of the observed associations between investor characteristics and investment performance.

**Table 6:** Ordered probit regression results with PSM method: robustness analysis.

Variables	Model 1	Model 2	Model 3	Model 4	Model 5	Model 6	Model 7
Financial advice_ from outsourced IFA of securities company	−0.0204	–	–	–	–	–	–
	(0.0202)						
Financial advice_ free from securities company	–	−0.0563∗∗∗	–	–	–	–	–
		(0.0099)					
Financial advice_ other securities companies	–	–	0.0409∗∗	–	–	–	–
			(0.0171)				
Financial advice_ financial experts apart from securities companies	–	–	–	−0.0184	–	–	–
				(0.0147)			
Financial advice_ mass media	–	–	–	–	−0.0146∗	–	–
	–	–	–	–	(0.0087)	–	–
Financial advice_ social media	–	–	–	–	–	−0.2303∗∗∗	–
	–	–	–	–	–	(0.0071)	–
Financial advice_ personal networks	–	–	–	–	–	–	−0.0420∗∗∗
							(0.0103)
Age	0.0329∗∗∗	0.0318∗∗∗	0.0321∗∗∗	0.0315∗∗∗	0.0352∗∗∗	0.0294∗∗∗	0.0325∗∗∗
	(0.0028)	(0.0025)	(0.0027)	(0.0027)	(0.0023)	(0.0021)	(0.0025)
Age squared	−0.0002∗∗∗	−0.0002∗∗∗	−0.0002∗∗∗	−0.0002∗∗∗	−0.0002∗∗∗	−0.0002∗∗∗	−0.0002∗∗∗
	(0.0000)	(0.0000)	(0.0000)	(0.0000)	(0.0000)	(0.0000)	(0.0000)
Male	−0.0580∗∗∗	−0.0782∗∗∗	−0.0718∗∗∗	−0.0835∗∗∗	−0.0812∗∗∗	−0.0738∗∗∗	−0.0871∗∗∗
	(0.0110)	(0.0098)	(0.0108)	(0.0106)	(0.0094)	(0.0074)	(0.0098)
University degree	−0.0200∗	−0.0175∗	−0.0223∗∗	−0.0237∗∗	−0.0248∗∗∗	−0.0026	−0.0085
	(0.0107)	(0.0097)	(0.0106)	(0.0104)	(0.0092)	(0.0075)	(0.0096)
Urban residency	−0.0130	−0.0141	−0.0106	−0.0190	0.0001	−0.0011	−0.0067
	(0.0134)	(0.0122)	(0.0131)	(0.0128)	(0.0112)	(0.0095)	(0.0119)
unemployed	0.0545∗∗	0.0391∗	0.0253	0.0381∗	0.0386∗∗	0.0744∗∗∗	0.0418∗
	(0.0232)	(0.0204)	(0.0220)	(0.0220)	(0.0186)	(0.0177)	(0.0216)
Married	0.0328∗∗	0.0358∗∗∗	0.0331∗∗	0.0371∗∗∗	0.0421∗∗∗	0.0537∗∗∗	0.0308∗∗∗
	(0.0132)	(0.0120)	(0.0130)	(0.0128)	(0.0114)	(0.0093)	(0.0119)
Have children	0.0029	0.0063	0.0104	0.0098	0.0013	−0.0111	−0.0008
	(0.0121)	(0.0110)	(0.0119)	(0.0117)	(0.0103)	(0.0086)	(0.0109)
Financial literacy	−0.2122∗∗∗	−0.2295∗∗∗	−0.2227∗∗∗	−0.2405∗∗∗	−0.2415∗∗∗	−0.2651∗∗∗	−0.1973∗∗∗
	(0.0163)	(0.0150)	(0.0162)	(0.0159)	(0.0145)	(0.0119)	(0.0141)
Log of household income	−0.1531∗∗∗	−0.1645∗∗∗	−0.1599∗∗∗	−0.1588∗∗∗	−0.1578∗∗∗	−0.1366∗∗∗	−0.1548∗∗∗
	(0.0104)	(0.0093)	(0.0100)	(0.0099)	(0.0087)	(0.0075)	(0.0093)
Log of household assets	−0.2372∗∗∗	−0.2363∗∗∗	−0.2369∗∗∗	−0.2335∗∗∗	−0.2385∗∗∗	−0.2285∗∗∗	−0.2213∗∗∗
	(0.0055)	(0.0049)	(0.0054)	(0.0053)	(0.0046)	(0.0040)	(0.0050)
Risk aversion	−0.0139	0.0155	0.0079	0.0028	0.0050	0.0103	0.0155
	(0.0205)	(0.0190)	(0.0202)	(0.0200)	(0.0179)	(0.0150)	(0.0189)
Myopic view of the future	0.0127	0.0080	0.0132	0.0105	0.0165	0.0009	0.0186
	(0.0131)	(0.0121)	(0.0130)	(0.0128)	(0.0115)	(0.0094)	(0.0117)
/cut1	−6.4475∗∗∗	−6.7066∗∗∗	−6.5832∗∗∗	−6.5560∗∗∗	−6.5690∗∗∗	−6.2375∗∗∗	−6.2995∗∗∗
	(0.1548)	(0.1398)	(0.1505)	(0.1482)	(0.1310)	(0.1124)	(0.1384)
/cut2	−4.6159∗∗∗	−4.8214∗∗∗	−4.7538∗∗∗	−4.7209∗∗∗	−4.7057∗∗∗	−4.3018∗∗∗	−4.3922∗∗∗
	(0.1535)	(0.1386)	(0.1492)	(0.1469)	(0.1299)	(0.1115)	(0.1373)
/cut3	−4.1388∗∗∗	−4.3488∗∗∗	−4.2825∗∗∗	−4.2529∗∗∗	−4.2327∗∗∗	−3.8681∗∗∗	−3.9126∗∗∗
	(0.1532)	(0.1383)	(0.1489)	(0.1466)	(0.1296)	(0.1113)	(0.1370)
/cut4	−3.6931∗∗∗	−3.8926∗∗∗	−3.8370∗∗∗	−3.8059∗∗∗	−3.7865∗∗∗	−3.4525∗∗∗	−3.4652∗∗∗
	(0.1530)	(0.1381)	(0.1487)	(0.1464)	(0.1294)	(0.1112)	(0.1368)
Observations	54,155	67,128	56,079	57,848	75,875	113,016	68,238
Log-likelihood	−61467	−74436	−63624	−65439	−84704	−118429	−74740
Chi2 statistics	4374	5357	4581	4725	6143	8914	4775
p-value	0	0	0	0	0	0	0

## 5 Discussion

The advisory functions of securities firms have faced increased competition amid the advent of new technology. Conventional professional financial advice has faced criticism over agency costs and moral hazards [[19], [20], [21], [22], [23], [24]]. However, a certain group of investors still rely on professional financial advice owing to financial knowledge and risk propensity [32,33,39]. By contrasting professional and free financial advisory services with subjective investment performance, we contribute new insights to the ongoing debate surrounding the need for costly financial advice.

Univariate analysis reveals that most investors rely primarily on free advisory sources such as social media, mass media, personal networks, and their judgment for investment decision-making. Seeking advice from professionals, including securities companies where investors hold accounts, is notably low. Interestingly, there is significant variation in the use of financial advice across different age groups: younger and middle-aged investors leaned more toward social media than their older counterparts, whereas older investors showed greater reliance on mass media compared with the younger and middle-aged groups. Additionally, in all age groups, a common trend was observed where investors tended to rely less on professional financial advice, particularly those involving fees. These findings align with those of Lal et al.’s [1], which highlight a shift in investor reliance on non-traditional financial information sources such as social media, blogs, and influencers over conventional sources such as professional advice or mass media. The inclination of older investors toward professional financial advice, although not very significant, could stem from their trust in account-holding companies or their reluctance to make independent decisions. This finding is also consistent with previous studies [71,73]. Kim et al. [73] found that older people with greater financial literacy, increased wealth, and complex financial circumstances tend to seek financial advice from professionals. Additionally, Korniotis and Kumar [71] noted that older investors tend to favor traditional information sources, leaning toward a rule-of-thumb approach.

The regression analysis reveals a notable association between various sources of financial advice and subjective investment performance. Investors who relied on free sources of financial advice such as free financial advice from the account-holding securities company, mass media, and social media experienced a significant profit in their investment accounts. However, reliance on professional financial advice from the account-holding securities company, other securities companies, and financial experts other than securities companies resulted in significant losses or failed to contribute significantly to their investment accounts. These findings are consistent with studies suggesting that seeking professional financial advice does not necessarily improve portfolio performance [[17], [18], [19],^23^]. Although agency costs could still be a problem for account-holding securities companies for subpar investment [22,23], the availability of alternative sources of financial advice is also responsible for investors’ reluctance to seek professional financial advice.

Although our study did not investigate the causes behind the poor investment performance of professional advice, we argue that when investors pay for professional advice, their expectations about investment results naturally increase. Consequently, disillusionment sets in if these expectations are not met and investors perceive their investments as failures. By contrast, investors seeking financial advice from social media, mass media, and personal networks reported significant profits in their investments. Information from these sources can be obtained free of charge. The results suggest that free financial advice obtained from online platforms can generate profitable investments. We argue that the emergence of alternative online information sources, particularly the advent of AI-based recommendations, significantly influences investment decision-making. However, our study could not provide additional evidence on how investor financial capabilities correlate with the use of online sources. Future research should investigate whether enhanced investment performance stems from investors’ financial expertise, exploring the potential link between their knowledge and superior investment outcomes.

In addition to the preference for free sources of financial advice, we identified distinct demographic and socioeconomic characteristics associated with the superior performance of investment portfolios. For instance, males, along with younger investors, those who are employed, unmarried, with children, possess financial literacy, and have higher income and household asset balances, tended to exhibit better performance in their portfolios. Our findings indicate that males exhibit better performance than females, which can be attributed to several factors, including differences in risk tolerance and investment behavior. Studies have demonstrated that men are generally more willing to take risks in their investment decisions, which can lead to higher returns and better performance [74]. Additionally, men may have more confidence in their financial knowledge and decision-making abilities, which can positively impact their investment outcomes [75]. Younger investors perform better in terms of minimizing losses. This can be explained by their longer investment horizon, which enables them to recover from short-term market fluctuations and benefit from the compounding effect of returns over time [76]. Younger investors are also more likely to be open to learning and adapting to new investment strategies, which can enhance their performance [77].

Among the socio-economic variables, employed individuals generally exhibit better investment performance. Employment provides a stable source of income, which can reduce the need to liquidate investments during market downturns and enable a more consistent investment strategy [78]. Additionally, employed individuals may have access to employer-sponsored retirement plans and investment education programs, which can improve their financial literacy and investment outcomes [79]. Individuals with children also perform well in investment performance. Parents often exhibit a higher level of financial responsibility and engage in long-term planning to provide for their children's future. This can lead to more prudent investment decisions, focusing on stability and growth. Studies have demonstrated that parents are motivated to invest in ways that will secure their children's future, such as funding education and other enrichment activities. These can translate into better financial management and investment performance [80]. Additionally, the need to provide a financial safety net for their children can drive parents to diversify their investments and avoid high-risk ventures, thereby reducing the likelihood of experiencing significant losses [81].

Unmarried individuals tend to perform better in investment performance because they have fewer financial responsibilities and obligations, enabling them to take on more risk in their investment portfolios [82]. Additionally, unmarried individuals may have more time and resources to manage their investments, which can lead to better performance [83]. Financial literacy is a significant factor in investment performance. Individuals with higher financial literacy are better equipped to understand and evaluate investment options, leading to more informed and effective decision-making [3]. Financially literate individuals are also less likely to be influenced by cognitive biases and emotional reactions, which can negatively impact investment performance [84]. Finally, the reason behind higher income and household asset balances and better investment performance could be derived from the fact that individuals with higher income have more disposable income to invest, enabling them to diversify their portfolios and reduce risk [5]. Higher household asset balances provide a financial cushion that can absorb potential losses and reduce the need to liquidate investments during market downturns [78]. Calvet et al. [85] also highlighted that households boasting higher incomes and assets are more prone to achieving enhanced investment performance.

The results of our study have important policy implications concerning the dominance of free financial advice over professional advice. First, enhancing financial literacy programs is crucial, as numerous investors increasingly rely on free sources such as social media and mass media for financial advice. These programs should equip individuals with the knowledge to critically assess the quality of the advice they receive, reducing the risks of poor investment decisions and misinformation. Additionally, regulating free advice platforms is essential to ensure that the information provided is accurate and transparent. Certification or vetting mechanisms for financial influencers and content creators could be introduced to improve the reliability of these sources. Furthermore, promoting hybrid advisory models that combine free and easily accessible advice with professional guidance could offer a cost-effective solution, enabling investors to benefit from both personalized expertise and the affordability of free advice. Moreover, policymakers should support the integration of AI-based robo-advisors, ensuring that these services are transparent and adhere to ethical standards to protect investors. Finally, the influence of free advice on market behavior, particularly the risks of herding behavior driven by social media, should be closely monitored to mitigate potential market distortions. These recommendations are designed to protect investors while recognizing the growing role of free financial advice in the current financial ecosystem.

This study has some limitations that warrant consideration when interpreting the results. First, our data exclusively involve internet-based customers, potentially affecting the generalizability of the sample. Second, we could not incorporate financial knowledge as a mediating variable when examining the relationship between financial advice and investment status. Third, owing to limitations in data availability, we could not measure investment performance objectively, preventing us from verifying the reliability of subjective investment evaluations, which could be affected by self-selection bias. Finally, our study did not explicitly include AI-based advisory services owing to limitations in our dataset. Nevertheless, we recognize that AI-driven robo-advisors are becoming significant in the free advisory ecosystem. Future research should consider integrating and comparing AI-based advice with traditional and free advisory services to gain a more comprehensive understanding of their relative impact on investment performance.

## 6 Conclusions

The recent advent of technology and the emergence of multiple sources of financial advisory services warrant the examination of how professional and free financial advice compete for profitable investments. Existing literature provides inconclusive evidence on the association between professional financial services and investment performance and a lack of evidence on whether free sources of financial advice contribute to superior investment performance. Using data from Rakuten Securities, we investigated the association between subjective investment status and professional and free financial advisory services. Our results show that more investors are inclined toward free sources of financial advice such as social media, mass media, and personal networks. Our regression results show that investors who used free financial advice from the account-holding securities company, social media, mass media, and personal networks achieved higher investment performance than those who used professional financial advice, particularly from the IFA of the account-holding securities company and financial experts from other securities companies. Among the control variables, males, along with younger investors, who are employed, unmarried, without children, possess financial literacy, and have higher income and household asset balances, tended to exhibit better performance in their portfolios.

Our findings have significant consequences for both potential investors and professional financial advisors. While previous studies have highlighted the underperformance associated with professional financial advice, our results further emphasize that investors may achieve better investment outcomes by utilizing free sources of financial advice. This highlights the necessity for professional financial advisors to demonstrate the added value and tangible benefits of their fee-based services, particularly in a landscape where free advisory sources are gaining prominence.

## CRediT authorship contribution statement

**Yoshihiko Kadoya:** Writing – review & editing, Validation, Supervision, Methodology, Funding acquisition, Formal analysis, Conceptualization. **Sumeet Lal:** Writing – original draft, Software, Methodology, Investigation, Formal analysis, Data curation. **Jin Shinohara:** Writing – original draft, Resources, Methodology, Investigation, Formal analysis, Data curation. **Mostafa Saidur Rahim Khan:** Writing – review & editing, Writing – original draft, Project administration, Methodology, Funding acquisition, Formal analysis.

## Ethical statement:

This study is based on a socio-economic survey, which does not involve any aspect of a direct human experiment. Moreover, all data used in this study are properly anonymized. The authors obtained informed consent from the participants to participate in the survey and publish the survey data in the study.

## Clinical trial

N/A.

## Data availability statement

Data has been uploaded as supplementary material.

## Funding

This work is supported by Rakuten Securities and 10.13039/501100001691JSPS KAKENHI (grant numbers JP24K21417, JP23K25534 and JP23K12503).

## Declaration of competing interest

The authors declare that they have no known competing financial interests or personal relationships that could have appeared to influence the work reported in this paper.
